# Delayed Revascularization in Rural Acute STEMI Patient on Direct Oral Anticoagulation: A Case Report

**DOI:** 10.5811/cpcem.64657

**Published:** 2026-10-20

**Authors:** Seth Roetman, Gary Brandecker

**Affiliations:** * Idaho College of Osteopathic Medicine, Meridian, Idaho; † Idaho College of Osteopathic Medicine, Department of Emergency Medicine, Meridian, Idaho

**Keywords:** ST-elevated myocardial infarction, rural, direct oral anticoagulation, percutaneous coronary intervention, case report

## Abstract

**Introduction:**

Acute ST-elevated myocardial infarction (STEMI) is a medical emergency requiring timely revascularization to preserve myocardial injury. Rural populations often experience delays in treatment leading to increased risk of complications and mortality. Emergent treatment of STEMI in a patient on long-term direct oral anticoagulation facing delays to revascularization is a complex scenario for rural physicians.

**Case Report:**

A 55-year-old male presented to the emergency department in rural Alaska and was diagnosed with acute inferior STEMI. The patient’s history was notable for pulmonary embolism with long-term direct oral anticoagulation. Thrombolytic therapy was not recommended and prompt revascularization via percutaneous coronary intervention (PCI) was needed; however, the nearest PCI-capable hospital was hundreds of miles away, increasing the risk of ischemic cardiomyopathy and death. The patient achieved revascularization after transport and, in the following year, experienced multiple episodes of ventricular tachycardia leading to ablation and need for implantable cardioverter-defibrillator placement.

**Conclusion:**

This case illustrates a complex but not uncommon scenario that rural physicians face. In rural patients requiring acute STEMI treatment but without timely access to a PCI-capable hospital, systemic thrombolysis and immediate transfer of care is often the only option for revascularization. There is a need for clear STEMI management guidelines in anticoagulated patients who present in rural settings.

## INTRODUCTION

Cardiovascular disease remains the most common cause of death in the United States and is expected to increase nationwide through 2050.^1^ Cardiovascular mortality is higher in rural areas compared to urban settings, with this disparity increasing after the coronavirus disease 2019 pandemic.^2^ In the case of ST-elevated myocardial infarction (STEMI), reperfusion therapy is recommended within 60 to 90 minutes of arrival at the hospital.^3^ Reperfusion is effective up to, and possibly beyond, 12 hours; however, necrosis and cell death of the myocardium increases with delays beyond the recommended 90 minutes, leading to an increased risk of life-threatening complications.^4^,^5^

The two methods of achieving reperfusion (or revascularization) are systemic thrombolysis or percutaneous coronary intervention (PCI). Percutaneous coronary intervention provides better outcomes and is the gold-standard treatment for acute STEMI.^6^ In rural patients requiring acute STEMI treatment but without timely access to a PCI-capable hospital, thrombolytics and immediate transfer is the next best option. The use of systemic thrombolysis in anticoagulated patients, however, carries a significant risk of bleeding and intracranial hemorrhage.^3^,^7^ In this case, we consider a man on long-term anticoagulation presenting with inferior STEMI in a rural area.

## CASE REPORT

A 55-year-old man with a history of persistent lower extremity deep vein thrombosis, pulmonary embolism, and uncontrolled hypertension presented to his local emergency department (ED) via ambulance with severe sternal chest pain, diaphoresis, and mild nausea. The patient’s chest pain began at approximately 7:45 am while exercising on a stationary bicycle. As the pain worsened he was brought to the hospital by emergency medical services.

Prehospital electrocardiography (ECG) demonstrated STEMI, which was confirmed on ECG obtained in the ED (Image 1). The patient was compliant with his rivaroxaban medication, which he was taking for a venous thromboembolism diagnosed three years prior.

Physical examination revealed the following vital signs: blood pressure, 147/100 mm Hg; heart rate, 47 beats per minute; and temperature, 98.2 ºF. Cardiac examination was notable for bradycardia, which was his baseline, with regular rhythm and normal first and second heart sounds present. The patient appeared diaphoretic and was clutching his chest with severe, sternal chest pain without radiation. In collaboration with the on-call cardiologist at the nearest PCI-capable hospital, it was decided that immediate transport was required. Due to the patient’s long-term oral anticoagulation, thrombolytic therapy was not recommended; thus, PCI was necessary for revascularization. An initial estimated time of arrival of 4.5 hours for medevac to the cardiac catheterization lab was given at 8:53 am (expected arrival at 1:23 pm).

*CPC-EM Capsule*

What do we already know about this clinical entity?

*Definitive treatment of ST-elevated myocardial infarction (STEMI) is percutaneous coronary intervention. When unavailable, systemic thrombolysis is the alternative, though it is often contraindicated.*

What makes this presentation of disease reportable?

*This case is notable for a rare combination of acute STEMI, lack of timely percutaneous coronary intervention access, transport delays, and ongoing anticoagulation complicating revascularization.*

What is the major learning point?

*Complex clinical decision-making is required in acute STEMI when transport is delayed and the patient is anticoagulated.*

How might this improve emergency medicine practice?

*In treating acute STEMI*,

*time is tissue.*

*Understanding potential barriers to management can improve patient outcomes.*

Patient management prior to transport included aspirin, acetaminophen, clopidogrel (600 mg), fentanyl, intravenous (IV) heparin (25,000 U), nitroglycerin, and intravenous fluids. The patient remained in the local hospital until transfer to medevac at 11:21 am and arrived at the PCI-capable hospital at approximately 2 am. More than six hours elapsed between arrival at the local ED and entry into the catheterization laboratory. The patient underwent a successful PCI and remained hospitalized for four days.

Follow-up over the next 18 months revealed an ejection fraction of 49.5% (50–70%), multiple near-syncopal episodes, and ventricular tachycardia (VT) during stress testing (Image 2). In a subsequent procedure, VT was easily induced in an area of inferoseptal scar resulting from the STEMI and ablated. As of this writing, the patient iwas scheduled to receive an implantable cardioverter-defibrillator.

## DISCUSSION

Rural living is increasingly attractive to many for a variety of reasons. However, it does not come without risks. Rural populations experience higher rates of cardiovascular disease and increased mortality.^2^ Rural communities face many barriers to healthcare including distance, terrain, and weather conditions that may make receiving preventive and acute care challenging.^8^,^9^ This frequently results in prolonged transport times during medical emergencies. This is especially worrisome in acute coronary syndromes, such as STEMI, because time to reperfusion is a significant determinant of patient outcomes.^3^,^10^ According to the American Heart Association (AHA), reperfusion therapy is recommended within 60 to 90 minutes of arrival at the hospital.^3^ Reperfusion is effective up to, and possibly beyond, 12 hours; however, cell death and necrosis of the myocardium increases with delays beyond the AHA-recommended time and leads to an increased risk of cardiovascular complications.^3^,^4^,^5^ Percutaneous coronary intervention is the gold standard treatment, leading to decreases in mortality and reinfarction compared to thrombolytics, the only other method of revascularization.^6^,^11^ However, many states have large rural populations without timely access to a PCI-capable hospital. This is true for over 40% of the population in 14 states including Alaska, Idaho, Iowa, Mississippi, New Mexico, and Oklahoma.^12^ Although the number of PCI-capable hospitals has recently increased, access among the US population has increased only marginally.^12^

When PCI is not immediately available, thrombolytic therapy is typically recommended.^3^ For rural patients requiring acute STEMI treatment, thrombolytics and immediate transfer of care to a PCI-capable hospital may be the only option. Thrombolytics are lifesaving, reducing mortality up to 24 hours from onset of symptoms.^3^ However, there are many contraindications to thrombolytics including bleeding disorders, recent head trauma, advanced liver disease, and a host of other relative contraindications.^3^ Current anticoagulant use is a relative contraindication according to the AHA guidelines for acute STEMI care.^3^ Previous evidence has shown that long-term oral anticoagulation with concurrent use of thrombolytics leads to a significantly higher risk of bleeding and intracranial hemorrhage.^3^,^7^ Recently, support has increased for concurrent use of direct oral anticoagulants and thrombolytics in other acute ischemic conditions.^13^,^14^ Based on the current recommendation, however, thrombolytic use is not an option in many cases.

Anticoagulant use is common and increasing. Oral anticoagulant use increased significantly from 2011 to 2019, nearly doubling from under three million Medicare beneficiaries to over five million.^15^ It is reasonable to assume these numbers have continued to increase in the last seven years as well; however, this does not account for those not covered by Medicare who require anticoagulation use.

## CONCLUSION

This case presents a unique but not uncommon scenario of a patient with an acute STEMI in a rural area facing delays to transport. The case was complicated further by the patient’s history of long-term direct oral anticoagulation. Acute STEMI is a medical emergency requiring immediate revascularization, typically achieved with PCI or thrombolytic therapy. Rural patients often lack timely access to PCI-capable hospitals and may be ineligible for thrombolytic therapy. This leaves the patient and physician in a complex and difficult situation. Proper management is imperative to minimize the risk of complications and death due to prolonged cardiac ischemia. Potentially broadening the use of thrombolytics in cases of delayed revascularization may be worth exploring. This case illustrates the need for clear management guidelines in acute STEMI care in a patient receiving long-term anticoagulation who faces substantial delays in transport.

## Figures and Tables

**Image 1:**
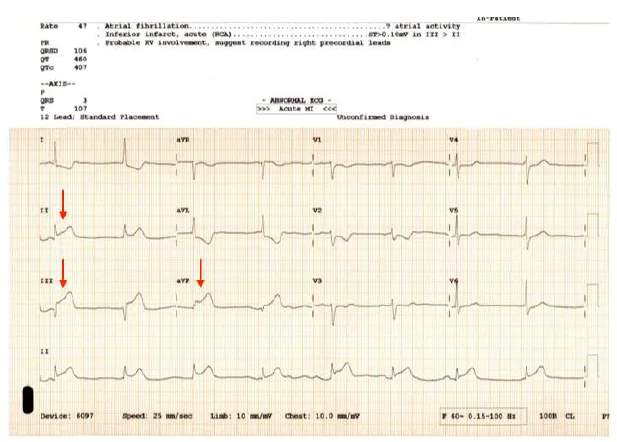
Electrocardiogram recorded in the local emergency department at 8:35 am demonstrating ST-elevation in leads II, III, and aVF, classic for an inferior myocardial infarction (arrows).

**Image 2:**
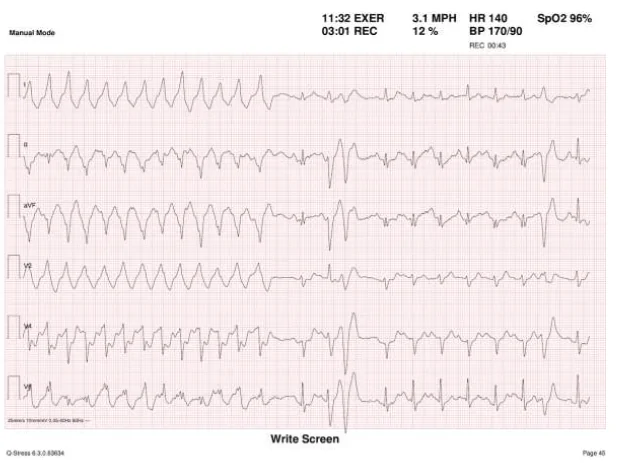
Stress electrocardiogram taken while incline walking on treadmill revealing ventricular tachycardia across multiple leads.
